# Pleural inhibition of the caspase-1/IL-1β pathway diminishes profibrotic lung toxicity of bleomycin

**DOI:** 10.1186/s12931-016-0475-8

**Published:** 2016-11-29

**Authors:** Olivier Burgy, Pierre-Simon Bellaye, Sebastien Causse, Guillaume Beltramo, Guillaume Wettstein, Pierre-Marie Boutanquoi, Françoise Goirand, Carmen Garrido, Philippe Bonniaud

**Affiliations:** 1 INSERM, LNC UMR866, LipSTIC LabEx team, Université Bourgogne Franche-Comté, 21000 Dijon, France; 2 Service de Pneumologie et Soins Intensifs Respiratoires, Centre Hospitalo-Universitaire de Bourgogne, 21000 Dijon, France; 3 Anticancer Centre Georges François Leclerc, CGFL, 21000 Dijon, France

**Keywords:** Idiopathic Pulmonary Fibrosis, Bleomycin, Pleural cells, Caspase-1, TGF-β1

## Abstract

**Background:**

Idiopathic and toxic pulmonary fibrosis are severe diseases starting classically in the subpleural area of the lung. It has recently been suggested that pleural mesothelial cells acquire a myofibroblast phenotype under fibrotic conditions induced by TGF-β1 or bleomycin. The importance and role of inflammation in fibrogenesis are still controversial. In this work, we explored the role of IL-1β/caspase-1 signaling in bleomycin lung toxicity and in pleural mesothelial cell transformation.

**Methods:**

C57BL/6 mice were intravenously injected with either bleomycin or nigericin or NaCl as control. In vitro, the Met5A cell line was used as a model of human pleural mesothelial cells.

**Results:**

Intravenous injections of bleomycin induced lung fibrosis with histologically-proven peripheral distribution, collagen accumulation in the pleural and subpleural area, and overexpression of markers of myofibroblast transformation of pleural cells which migrated into the lung. These events were associated with an inflammatory process with an increase in neutrophil recruitment in pleural lavage fluid and increased caspase-1 activity. TGF-β1 was also overexpressed in pleural lavage fluid and was produced by pleural cells following intravenous bleomycin. In this model, local pleural inhibition of IL-1β with the IL-1β inhibitor anakinra diminished TGF-β1 and collagen accumulation. In vitro, caspase-1 inhibition interfered with Met5A cell transformation into the myofibroblast-like phenotype induced by bleomycin or TGF-β1. Moreover, nigericin, a caspase-1 activator, triggered transformation of Met5A cells and its intra-pleural delivery induced fibrogenesis in mice.

**Conclusions:**

We demonstrated, after intravenous bleomycin injection in mice, the role of the pleura and highlighted the key role of IL-1β/caspase-1 axis in this fibrogenesis process.

**Electronic supplementary material:**

The online version of this article (doi:10.1186/s12931-016-0475-8) contains supplementary material, which is available to authorized users.

## Background

Idiopathic Pulmonary Fibrosis (IPF) is a devastating disease characterized by matrix accumulation in the lung leading to death with a mean survival of three to 5 years after diagnosis [1]. To date, the causes of IPF remain elusive and the therapeutic options available to patients are limited, with only two drugs recently approved, pirfenidone and nintedanib, both of which have demonstrated only a slight impact on disease progression [1–3]. IPF typically starts from subpleural areas of the lung and then progresses deeper towards the lung parenchyma, where it disturbs alveolar architecture and gas exchange. Bleomycin (BLM) is an effective chemotherapeutic agent widely used intravenously in humans mainly in patients with Hodgkin’s lymphoma. However, the pulmonary toxicity of BLM has restricted its use in clinical practice (www.pneumotox.com).

The role of mesothelial cells in animal models of pulmonary fibrosis and in human IPF has recently been reported [4–11]. In these diseases, they differentiate into myofibroblast-like cells via a cellular mechanism called Mesothelio-Mesenchymal Transition (MMT) [5]. However, the exact mechanisms that lead to the involvement of the pleura and pleural mesothelial cells in the onset and progression of fibrosis are not yet fully understood.

The role of chronic inflammation in human IPF is still controversial. Inflammatory cytokines and the infiltration of immune cells are found in IPF [12, 13]. IL-1β overexpression in rodent lung induced the upregulation of TGF-β1, a major pro-fibrotic growth factor, and lung fibrosis [14, 15]. IL-1β is synthesized as a latent form and is activated following cleavage by caspase-1. Procaspase-1 is activated in multiprotein complexes, called the inflammasome, which contains a NOD-like receptor and a scaffold protein such as the apoptosis associated speck-like protein containing a CARD (ASC). The importance of IL-1β/caspase-1 signaling has been shown in the BLM model of lung fibrosis [16]. Mice deficient for inflammasome components such as the NOD-like receptor NLRP3 and thereby unable to activate caspase-1 and thus produce active IL-1β develop less severe fibrosis after BLM challenge [17]. Even though most studies on IL-1β/caspase-1 signaling focused on immune cells, some studies highlighted the involvement of this signaling pathway in structural cells [18, 19]. The role of caspase-1 on lung structural cells and its involvement during fibrotic processes is still poorly understood.

In the present study, using repetitive intravenous injections of BLM in mice, we highlighted the pleural activation of IL-1β/caspase-1 signaling as a seminal event in BLM-induced pleural and subpleural fibrotic toxicity. Our work suggests that caspase-1 could be a therapeutic target for IPF as well as BLM lung fibrotic toxicity.

## Methods

### Animal procedures

Eight-week-old C57BL/6J mice (Charles River, Saint Germain-sur-l’Arbresle, France) were housed accordingly to the guidelines of the *“Ministère de la Recherche et de la Technologie”*. All experiments were approved by the *“Comité d’Ethique de l’Expérimentation Animale (C2EA) du grand campus Dijon, n°105”* (ref number : 4612). Mice were intravenously injected three times per week with bleomycin (BLM. Calbiochem) at a dose of 20 mg/kg for a total of 6 injections (Additional file 1: Figure S1A). Il-1β signaling was blocked with IL-1ra (anakinra) that was injected (0.1, 1 or 5 mg) intra-pleura every other day from day 0 to day 14 (Additional file 1: Figure S1B). Caspase-1 was activated by intrapleural injection of nigericin (Sigma Aldrich). Three, 14 or 21 days after the beginning of the injections and after a sample of blood was collected, mice were euthanized by abdominal aortic bleeding. Bronchoalveolar lavage fluid (BALF) and pleural lavage fluid (PLF) were then collected as previously described [5]. For histological analysis, lungs were harvested, inflated and fixed in formalin. Intrapleural injection of β-Galactosidase coding adenoviruses (AdLacZ) were administered as previously described [4, 5]. After being harvested, the lungs were placed in a solution containing β-Galactosidase substrate. Hydrolysis of this substrate released a blue-colored product highlighting pleural adenovirus-infected cells.

### Histology and fibrosis assessment

ibrosis in pleural and subpleural areas was assessed on lung sections after H&E staining using a scoring method based on the modified Ashcroft score previously described [20]. Lung sections were analyzed in a double-blinded test to assess the pleural area using ×100 magnification and were graded from 0 (normal lung) to 8 (completely fibrotic lung). When varying degrees of fibrosis were present, the highest score was retained. Collagen was quantified using a histomorphometric assay on picrosirius red stained sections [5]. Ten areas targeting the pleura were randomly acquired under polarized light (×100. Nikon Eclipse E600) with a high-resolution microscope camera (Nikon DS-Ri1). Signal intensity was quantified using a homemade ImageJ macro and plotted as signal intensity according to distance to the pleura (up to 500 μm into the lung parenchyma) (Additional file 2: Figure S2). Briefly, the user inputs a pleura limit, a parenchymal side (above, below, left, right), a background area and areas to exclude from the analysis (staining artifacts, vessels and large airways). The program adjusts the pleural limits, the beginning of the pleura being set as the first of two consecutive pixels (in an order from the background to the parenchyma) with an intensity higher than the mean background signal plus two standard deviations from the background. From there on, the program maps the distance from the parenchymal area to the pleura (Additional file 2: Figure S2A). It then measures the intensity of each parenchymal pixel in the picrosirius red channel. The program returns a text file with the intensity and distance to pleura of each measured pixel. The data are plotted to form a curve with the mean intensity of all pixels with a given distance to the pleura (Additional file 2: Figure S2B). Statistical analyses were performed by comparing areas under the curve over the whole range in the pleura (0–60 μm) or subpleural (60–500 μm) area. Picrosirius red was also monitored in the parenchymal area as previously described [21]. For collagen quantification, vessels and large airways were excluded. Total TGF-β1 levels in BALF or PLF were assessed with a specific ELISA for mouse TGF-β1 (Quantikine® ELISA. R&D Systems) according to the manufacturer’s guidelines.

### Cell culture

Human pleural mesothelial cells Met5A were cultured in Medium-199 (Lonza) supplemented with 10% serum (Hyclone, Fisher Scientific). Proliferating cells were treated with BLM (Calbiochem). For Il-1β assessment in supernatant, cells were treated with BLM in serum-free medium. When indicated, caspase-1 was blocked using YVAD (Bachem AG) as previously described [19].

### Immunohistology

Formalin fixed, paraffin embedded lung sections were dewaxed with xylene and endogenous peroxidases were inhibited (H_2_O_2_ 3%). Frozen sections were fixed with ice-cold acetone and cells fixed with PFA and permeabilized with triton. Before saturation (BSA 5%), samples were incubated overnight at 4 °C with specific antibody. HSP47 (LF-PA41903) was from Ab Frontier, WT-1 (ab89901) from Abcam, Ki-67 (PA1-21520) from Thermo, HSP27 (SPA-803) and αB-crystallin (SPA-223) from Enzo Life Sciences, α-SMA (ab5694) from Abcam and NLRP3 (Cryo-2) from Adipogen. After washing, sections were incubated with HRP-coupled anti-rabbit antibody (Jackson Immunoresearch Laboratories) or Alexa568- or Alexa488-coupled antibody (Invitrogen). NovaRED (Vector Labs) was used to detect HRP followed by counterstaining with hematoxylin for immunohistochemistry. DAPI-containing ProLong® Gold (Invitrogen) was used for immunofluorescence experiments. For caspase-1 fluorescent staining, frozen lung sections were labeled according to the manufacturer’s instructions (FAM-FLICA YVAD. ImmunoChemistry Technologies). TGF-β1 expression was followed by a FISH analysis on dewaxed sections. Briefly, sections were incubated in hybridization solution (Formamide 20%, SSPE 2X) and then incubated with a mix of Quasar 570-coupled probes (“Stellaris”, Biosearch Technologies) specifically targeting murine TGF-β1 mRNA. After incubation, excess was eliminated from the probes and sections were mounted. Images were acquired with a Nikon Eclipse E600 with a high-resolution microscope camera (Nikon DS-Ri1). Fluorescent images were visualized using an Axio Imager.M2 microscope (Zeiss).

### Western blot

Proteins from cells or lung tissues were extracted using a Triton X-100 and anti-protease (Roche) containing buffer for 30 to 40 min on ice. Proteins were quantified using a colorimetric method (Bio-Rad Protein Assay. Bio-Rad) before thermic denaturation in loading buffer containing SDS and β-mercaptoethanol. For Il-1β assessment, supernatants were collected and centrifuged to pellet debris. Samples were concentrated using a methanol-chloroform method before denaturation. To evaluate active MMP expression, a non-denaturant loading buffer was used to deposit BALF or PLF for the SDS-PAGE. 30 μg of proteins for lysates or constant volume for supernatant were deposed for migration on 12–15% acrylamide gels according to the molecular weight of the targeted proteins. After electrophoresis, proteins were transferred on to PVDF membranes (GE Healthcare Europe GmbH) in a boric acid containing buffer. For IL-1β transfer, an ethanol-supplemented glycin buffer was used. Membranes were saturated with BSA before incubation with primary antibodies. MMP-9 (BML-SA620) from Enzolife Science, MMP-2 (sc-13594) and HSC70 (clone B-6) from Santa Cruz Biotechnology, HSP47 (LF-PA41903) from Abfrontier, Caspase-1 (AHZ0082) from Invitrogen, Il-1β (clone 166926) from R&D, α-SMA (ab5694) from Abcam, E-Cadherin (24E10) from Cell Signaling and β-actin (AC-74) from Sigma-Aldrich. After washing in TBS-Tween 0.1%, membranes were incubated with HRP-coupled secondary antibody (Jackson Immunoresearch Laboratories). The signal was revealed using a chemiluminescent reagent (Western Blotting Luminol Reagent. Santa Cruz Biotechnology) and was followed by a ChemiDoc XRS System (Bio-Rad).

### RT-qPCR analysis

Total RNA from mouse lung was extracted using TRIzol (Invitrogen). Reverse transcription was performed on the total RNA using the M-MLV kit (Promega) and quantitative RT-PCR was performed on the cDNA using SYBR green master mix (Promega). The forward and reverse primer sequences were the following: for mouse samples: TGF-*β*1, 5′-CGTGGCTTCTAGTGCTGACGC-3′ and 5′-CCATGTCGATGGTCTTGCAGGT-3′; PAI-1, 5′-GGCCGTGGAACAAGAATGAGAT-3′ and 5′-GCTTGAAGAAGTGGGGCATGAAG-3′; E-cadherin, 5′-GGAGAGGCACCTGGAGAG-3′ and 5′-TCCGAAAAGAAGGCTGTC-3′; L32, 5′-GAAACTGGCGGAAACCCA-3′ and 5′-GGATCTGGCCCTTGAACCTT-3′, for human samples: E-cadherin, 5′-ACA GCC CCG CCT TAT GAT T-3′ and 5′-CTTCGGAACCGCTTCCTTCA-3′; α-SMA, 5′-TGGTCGGTATGGGTCAGAAAG-3′ and 5′-TCAGGGTCAGGATACCTCTCTTG-3′; procollagen, 5′-GCTACCCAACTTGCCTTCATG-3′ and 5′-GCAGTGGTAGGTGATGTTCTAAGA-3′; L32, 5′-TGTCCTGAATGTGGTCACCTGA-3′ and 5′-CTGCAGTCTCCTTGCACACCT-3′.

### Proteome profiler

Relative levels of several cytokines were assessed with the “Mouse Cytokine Array Panel A Array kit” (R&D Systems). Mouse plasma was tested as recommended by the manufacturer. Signal intensity was quantified by ImageJ software and data expressed as a percentage of intensity normalized to positive controls.

### Statistical analysis

All experiments were repeated at least three times independently. The different groups were compared using a two-tailed nonparametric unpaired Mann Whitney test. *P* values below 0.05 were considered statistically significant.

## Results

### Subpleural fibrosis induced by intravenous bleomycin administration is associated with pleural cell migration

**Fig. 1:**
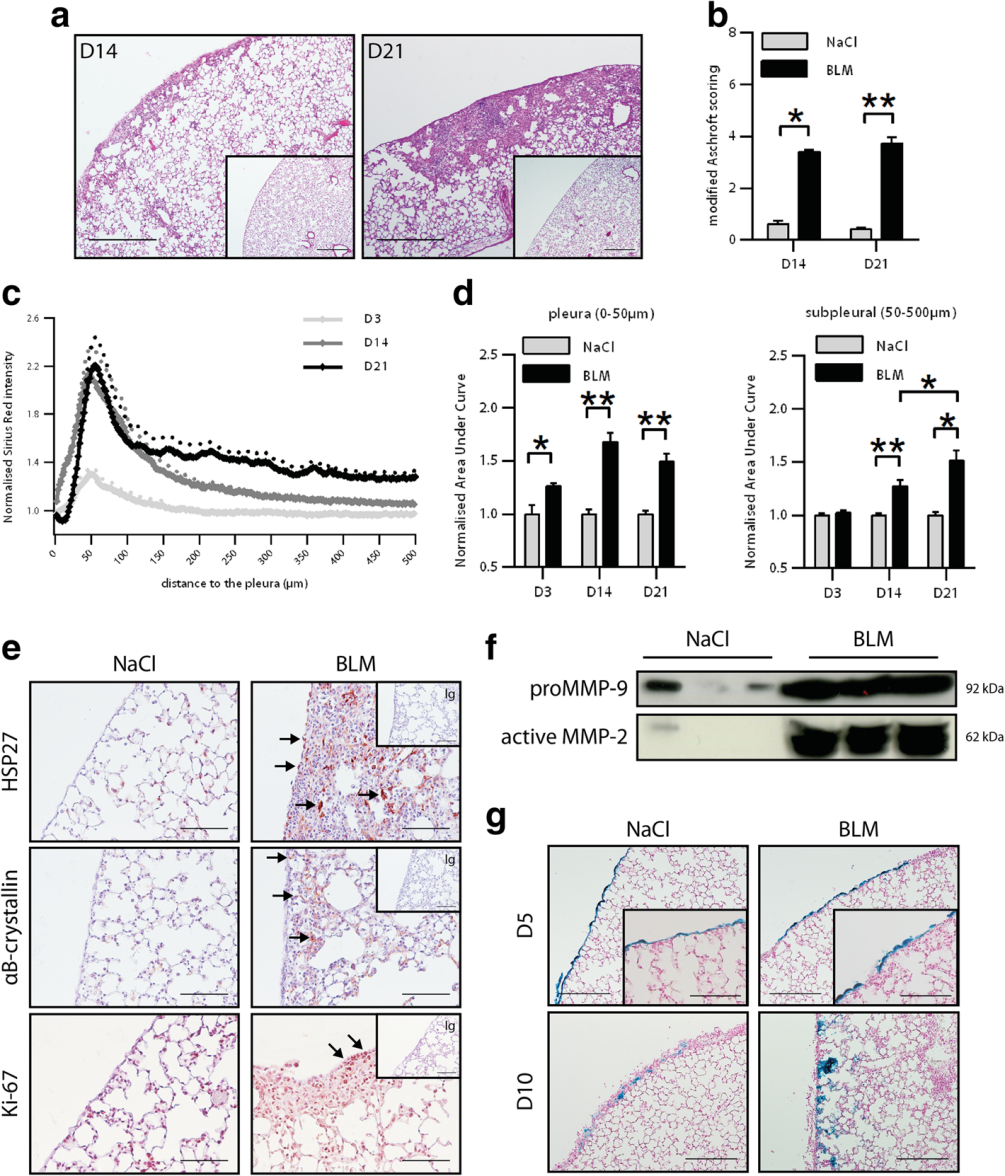
Intravenous bleomycin induces subpleural fibrosis associated with pleural cell differentiation and migration. **a** Histological analysis of lung sections from mice receiving either NaCl or BLM intravenously at D14 and D21. Representative images (insert: NaCl at the corresponding time point. Scale bars : 500 μm). **b** Ashcroft scoring of lung sections. **c** Profiles of Picrosirius Red signal according to distance to the pleura at D3, D14 and D21. **d** Area Under Curve comparison of areas targeting the pleura (*left*) or subpleural (*right*) areas at D3, D14 and D21. For collagen quantification, data are represented as mean of Picrosirius Red signal normalized to the NaCl condition at the corresponding time point. Data expressed as mean ± SEM. *n* = 4 for NaCl groups, *n* = 6 for BLM groups at D3 and D21, and *n* = 6 for NaCl and n = 8 for BLM at D14. **e** Representative images of immunostaining for HSP27, αB-crystallin and KI-67 of lung sections from mice receiving NaCl or BLM at D14 (arrows indicate positive cells. Counterstaining: Harris hematoxylin. Scale bars : 100 μm). **f** Levels of proMMP-9 and active MMP-2 in PLF was determined by western blot. Representative results from NaCl (*n* = 3) or BLM (*n* = 3) treated mice at D14 are shown. **g** AdLacZ-labeled pleural cells were stained for β-galactosidase activity (*blue staining*) in lung section from mice receiving intravenously NaCl or BLM at D5 or D10. Representative images are shown (*n* = 4 NaCl, *n* = 6 BLM. Scale bars: 200 μm), Insert: magnification; scale bars: 100 μm Arrows indicate pleural cells with modified morphology. Counterstaining: Nuclear fast red). *p ≤ 0.05, **p ≤ 0.01

Histological analysis of lung sections, quantified by modified Ashcroft scoring and picorsirius red quantification, showed that repeated intravenous injections of bleomycin (BLM) induced progressive subpleural morphological changes in mouse lungs, compared with NaCl controls (Fig. 1a, Additional file 3: Figure S3A, B). By D14, morphological changes were mainly observed in the subpleural areas, and progressed towards the inner parenchyma by D21 (Fig. 1a, Additional file 3: Figure S3C–E). We set up a novel method to quantify picrosirius red staining by taking into consideration the distance to the pleura, and performed in-depth analyses (Additional file 2: Figure S2). We thus showed that, following intravenous administration of BLM, collagen started to accumulate in the pleura at D3 and progressed towards the lung parenchyma in a time-dependent manner (Fig. 1c, d). In line with this, in the lungs of BLM-treated mice but not in those of control mice, HSP47 was overexpressed in pleural cells as early as D3 and in fibrotic subpleural areas at D14 (Additional file 2: Figure S2F, G). Intravenous BLM promoted the upregulation of HSP27 and αB-crystallin in the fibrotic areas (Fig. 1e). These proteins have been reported to promote pleural mesothelial cell transformation [4, 9]. We also observed an increase in the expression of the marker of Ki-67 proliferation in pleural cells next to the fibrotic areas (Fig. 1e). Moreover, we detected an increase in proMMP-9 expression and MMP-2 activation in the pleural lavage fluid (PLF) from BLM-treated mouse lung, compared with the control group (Fig. 1f). We thus investigated the migration of pleural cells after intravenous BLM injection. Following intrapleural injection of LacZ-coding adenovirus (AdLacZ), which specifically labels pleural cells [5], we noticed a change in pleural cell morphology (arrows) at D5 in BLM-treated mice, compared with control mice (Fig. 1g). Furthermore, at D10 after the BLM injection, pleural cells were found within the subpleural lung parenchyma whereas in control animals the pleural cells remained in the pleural layer. These results demonstrate that systemic BLM injections trigger the transformation of pleural cells, which then migrate into the lung parenchyma.

### Pleural cells are involved in bleomycin-induced inflammation

**Fig. 2:**
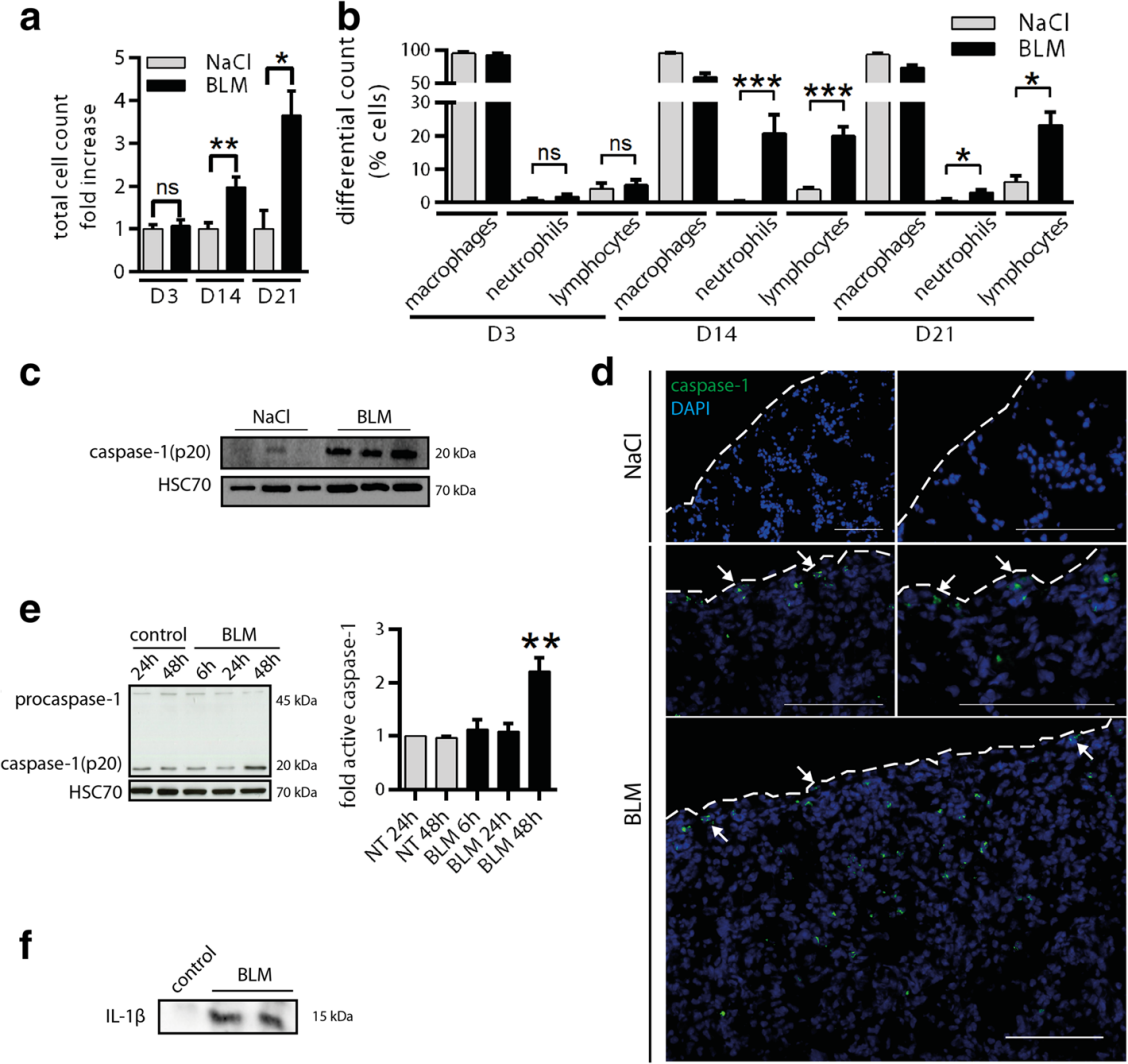
Pleural cells are involved in bleomycin-induced inflammation. **a** Cell recruitment in PLF was assessed by total cell count at D3, D14 and D21. Results are expressed as mean ± SEM. **b** Inflammation was estimated by differential count in PLF after May-Grünwald Giemsa staining. Results are expressed as percentages of total cells. *n* = 4 for NaCl groups, *n* = 6 for BLM groups at D3 and D21, *n* = 6 for NaCl and *n* = 8 for BLM at D14. **c**, **d** Caspase-1 activation was assessed at D14 by **c** western blot in whole lung extracts (*n* = 3 mice/group) or by **d** specific caspase-1 FLICA analysis on lung section from NaCl or BLM injected mice (Representative images are shown. Active caspase-1: green, DAPI: blue, dotted lines represent the pleura, scale bars: 50 μm). **e**, **f** Met5A cells were treated with BLM (100 μM) and **e** activation of caspase-1 was followed (western blot; left, densitometric analysis; right). **f** active IL-1β was assessed in the supernatant of Met5A cells after 24 h of culture. Representative results from 3 independent experiments. HSC70 serves as a loading control. *p ≤ 0.05, **p ≤ 0.01, ***p ≤ 0.001

Compared with controls, intravenous BLM induced an increase in the total number of cells in PLF starting at D14 (Fig. 2a). At D14 and D21 after the BLM systemic administration, we also found an increase in the percentage of neutrophils and leucocytes in the PLF (Fig. 2b). This pro-inflammatory profile at the pleural level was also found in the broncho-alveolar lavage fluid (BALF) following BLM (Additional file 4: Figure S4A, B). This suggests that the inflammatory response to the intravenous administration of BLM may involve the lung as well as the pleural area. Moreover, no differences were found in total cell count in the blood (monocytes, granulocytes, lymphocytes) or in serum levels of the major pro-inflammatory cytokines. (Additional file 4: Figure S4C, D). We then focused on the caspase-1/IL-1β pathway. Systemic administration of BLM in mice triggered the activation of caspase-1 in the lung in particular in the pleural and subpleural area (Fig. 2c, d). In the human pleural mesothelial Met5A cell line, BLM induced caspase-1 activation as well as active IL-1β secretion into the supernatant (Fig. 2e, f), and caspase-1/IL-1β activation correlated with the presence of NLPR3 aggregates, suggesting inflammasome formation (Additional file 5: Figure S5).

### Pleural delivery of IL-1ra hampers bleomycin-induced TGF-β1 upregulation

**Fig. 3:**
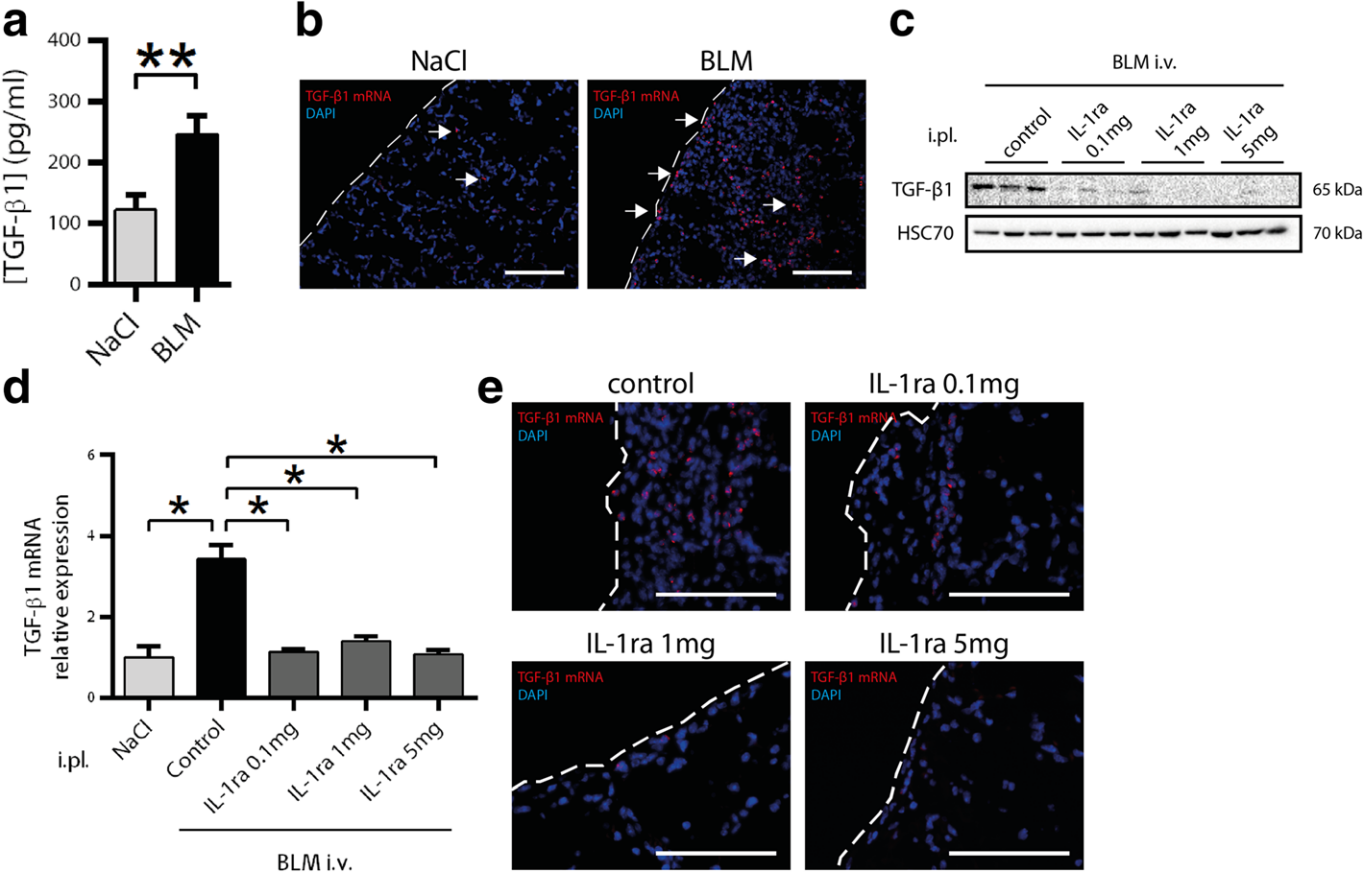
Pleural delivery of IL-1ra hampers bleomycin-induced TGF-β1 upregulation. **a** TGF-β1 accumulation in the PLF of NaCl- or BLM-injected mice at D14 was measured by ELISA. Results are expressed as mean ± SEM. *n* = 8 NaCl, *n* = 10 BLM. **b** Representative pictures from TGF-β1 mRNA expression analysis at D14 by FISH in lung sections from mice receiving NaCl (*n* = 4) or BLM (*n* = 6). TGF-β1 mRNA: red, DAPI: blue, scale bars: 50 μm. Pleura (*dotted line*) and TGF-β1 mRNA expression spots (*arrows*) are indicated. **c** TGF-β1 protein level assessed by western blot on whole lung from mice co-injected intravenously with NaCl or BLM together with intrapleural IL-1ra (from day 0 to 14) at the indicated doses or NaCl as a control at D21. HSC70 serves as a loading control, *n* = 3 mice/group. **d** qPCR analysis for the expression of TGF-β1 mRNA at D21. Expression relative to L32 and normalized with the NaCl group is shown, *n* = 5 mice/group. **e** FISH staining for TGF-β1 mRNA expression on lung sections from the above described mice co-injected with BLM and IL-1ra (*n* = 5 mice/group, scale bars: 50 μm). *p ≤ 0.05, **p ≤ 0.01

We observed at D14 that cells localizing in the pleura exhibited transformation features such as Ki-67 or HSP27/αB-crystallin overexpression. To investigate if pleural cells were also involved in the establishment of a profibrotic milieu, we investigated TGF-β1 production at this same time-point (e.g. D14). We observed an upregulation of TGF-β1 in the PLF from BLM-injected mice compared with controls (Fig. 3a). A Fluorescent *In Situ* Hybridization (FISH) analysis of lung sections showed that BLM treatment increased the expression of TGF-β1 mRNA in pleural cells whereas barely any expression was observed in control animals (Fig. 3b). A similar upregulation of TGF-β1 production was observed at D21 (Additional file 6: Figure S6). As inflammation is linked to TGF-β1 upregulation, we next investigated the relationship between BLM-induced IL-1β production by pleural cells and fibrosis markers such as TGF-β1 upregulation. Repeated intra-pleural administration of a specific inhibitor of IL-1β (IL-1ra) during the inflammation step (from day 0 to day 14) decreased TGF-β1 induction in the lungs (both mRNA and protein) induced by the intravenous administration of BLM at D21 (Fig. 3c, d). FISH analysis showed that the injection of IL-1ra was able to inhibit TGF-β1 expression in both pleural cells and cells localized in subpleural areas (Fig. 3e).

### Pleural IL-1ra prevents bleomycin-induced lung fibrosis

**Fig. 4:**
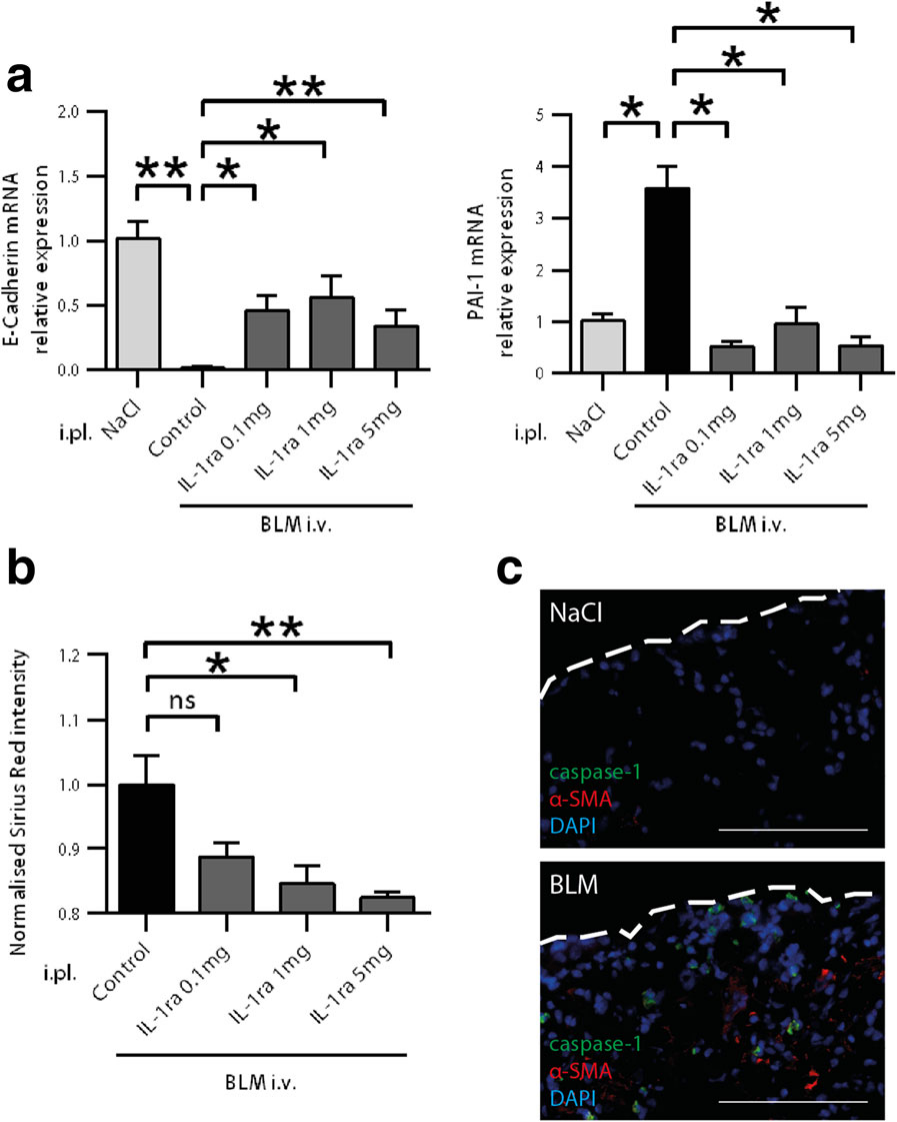
Pleural IL-1β/caspase-1 inhibition interferes with bleomycin-induced lung fibrosis. Mice were injected intravenously with NaCl or BLM together with intrapleural delivery of IL-1ra at the indicated doses or NaCl as a control (*n* = 5 mice/group). **a** qPCR analysis for the expression of E-Cadherin and PAI-1. Expression relative to L32 and normalized with the NaCl group is shown. **b** Histomorphometric quantification of collagen in lung parenchyma on lung sections from the above described mice. **c** Double staining for FLICA caspase-1 (*green*) and α-SMA (*red*) on lung sections of mice intravenously injected with BLM or NaCl. Representative images, *n* = 5/group. Nuclear staining: DAPI (*blue*), dotted lines represent the pleura, scale bars: 50 μm. *p ≤ 0.05, **p ≤ 0.01

We studied whether the intra-pleural delivery of IL-1ra interfered with BLM pro-fibrotic toxicity in the lung. Pleural delivery of IL-1ra increased expression levels of E-Cadherin and decreased those of PAI-1 (Fig. 4a). In line with this finding, IL-1ra reduced collagen accumulation in mouse lung following BLM injection in a dose-dependent manner as observed by histomorphometric analysis (Fig. 4b). Further, as shown in Fig. 4c, in BLM-injected mice compared with the NaCl control, α-SMA was overexpressed in lung areas where caspase-1 was activated. Altogether, these data suggest that caspase-1/IL-1β signaling is involved in the transformation process occurring in the subpleural area, which represents a triggering event in fibrogenesis.

### Caspase-1 inhibition hampers pleural structural cell differentiation

**Fig. 5:**
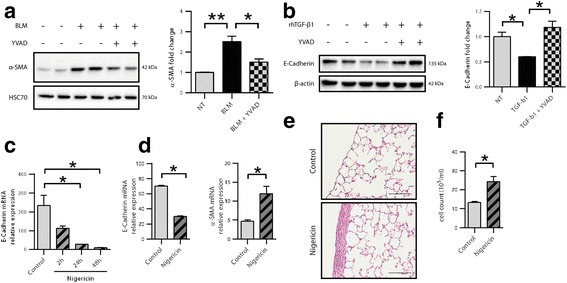
Caspase-1 inhibition hampers pleural structural cell transformation. **a**, **b** Met5A cells were cultured with **a** BLM or **b** TGF alone or in combination with caspase-1 inhibitor YVAD (western blot; left, densitometric analysis; right). Expression of α-SMA and E-Cadherin were assessed by western blot. HSC70 and β-actin: loading control. Representative results from three independent experiments. **c**, **d** qPCR analysis of E-Cadherin and α-SMA mRNA on Met5A cells cultured in the presence of nigericin (0.1 μM) or control **c** at the indicated times or **d** for 24 h. C57Bl/6 mice were given intrapleural injections of nigericin (1.25 mg/kg) or control. **e** Histological analysis at D21 post-intrapleural injection. Representative observations after H&E staining, scale bars: 100 μm. **f** Total cell count in the PLF at D14 after intrapleural injection of nigericin or control. *p ≤ 0.05, **p ≤ 0.01

We then investigated the role of caspase-1 in pleural cell differentiation in vitro. In the presence of BLM, Met5A cells exhibited a mesenchymal phenotype with the overexpression of α-SMA (Fig. 5a). The specific caspase-1 inhibitor YVAD was able to counteract these changes. Furthermore, YVAD also abolished the E-Cadherin downregulation induced by TGF-β1 (Fig. 5b). To further confirm the involvement of caspase-1 in the process of pleural cell transformation, Met5A cells were cultured in the presence of the caspase-1 activator nigericin. As shown in Fig. 5c, E-cadherin expression gradually decreased as caspase-1 was activated. Compared with the control, nigericin induced other MMT-like changes such as α-SMA overexpression (Fig. 5d). In the same way, intra-pleural delivery of nigericin in mice induced a fibrotic response in the pleura (Fig. 5e). Moreover, when injected into the pleura, nigericin induced an increase in cell recruitment in the pleura of these animals (Fig. 5f). Collectively, these data indicate that caspase-1 is involved in the activation and subsequent transformation of pleural cells triggered by BLM or TGF-β1.

## Discussion

In recent decades, chronic inflammation has been considered a non-essential factor in the development of IPF. However, there is evidence that inflammation plays a role not only at the onset of the fibrotic process but also during fibrosis progression [22]. IPF progression is interspersed with stable periods interrupted by acute exacerbation, in which TGF-β1 plays a key role [23, 24]. The cause of these exacerbations remains unknown, though inflammation is thought to be a triggering event [25]. A retrospective study highlighted the beneficial effect of soluble thrombomodulin on mortality in patients with an acute exacerbation of IPF [26]. Interestingly, thrombomodulin, besides its role in the modulation of intravascular coagulation, also inhibits HMGB1, a trigger of the NLRP3 inflammasome and subsequent caspase-1 activation [27]. The involvement of caspase-1/IL-1β in BLM-induced lung toxicity has already been reported in animal models [16, 17]. Recently, structural cells have been reported to be involved in capsase-1 activation and IL-1β secretion in the lung following different profibrotic stimuli [18, 19]. Mesothelial cells have already been shown to be involved in the initiation of the inflammatory response in a model of talc-induced pleurodesis [28]. Furthermore, asbestos can induce caspase-1 activity through the NLRP3 inflammasome and thus triggers an inflammatory signal in mesothelial cells [29]. Our work highlighted the fact that local intrapleural treatment with an IL-1β inhibitor such as IL-1ra (to counteract BLM-induced pleural inflammation) limited fibrosis progression and could thus be a therapeutic option notably in the management of acute exacerbation of IPF. It worth to note that IL-1ra effect on established fibrosis still needs to be investigated.

We and others have demonstrated the involvement of pleural mesothelial cells in the process of pleuro-pulmonary fibrosis in animal models as well as in human IPF [5, 6, 8]. We previously described in several animal models that pleural mesothelial cells can differentiate into myofibroblast-like cells under TGF-β1 through a transformation process called MMT [4–6, 9]. Interestingly, recent studies revealed the presence of calretinin and mesothelin expressing cells, two markers of pleural mesothelial cells, in IPF lung parenchyma suggesting the migration of pleural mesothelial cells [7, 8, 30]. Our present work further endorses these results as we described the differentiation and migration of pleural cells together with increased collagen production after systemic administration of BLM. Moreover, we provide evidence regarding the direct role of caspase-1 in BLM- and TGF-β1-induced pleural cell transformation. NLRP3 has been reported to interfere with TGF-β signaling [31, 32]. NLRP3 can regulate cardiac fibroblast differentiation, and its deficiency seems to protect mice from angiotensin II-induced cardiac fibrosis [31]. NLRP3 hampers TGF-β–induced EMT in vitro in renal tubular epithelial cells [32]. In line with these findings, we showed here that activation of caspase-1 was able to trigger transformation/differentiation of pleural cells in vitro. Moreover, intra-pleural delivery of nigericin in mice elicits fibrogenesis with a restrictive pleural localization at D21, suggesting that pleural activation of caspase-1 could induce pleural cells transdifferentiation. Supporting this hypothesis, caspase-1 inhibition inhibited transdifferentiation of Met5A cells induced by either BLM or TGF-β1. Thus, targeting caspase-1 in pulmonary fibrotic disorders might represent an interesting therapeutic option by interfering with two pro-fibrotic events: inflammation and the transformation of pleural structural cells that acquire a myofibroblast-like phenotype.

## Conclusion

Using a murine model of pulmonary fibrosis induced by repetitive intravenous injections of BLM, which corresponded to a robust experimental model of pulmonary fibrosis mimicking human IPF and human BLM-induced lung toxicity, we confirmed the direct role of pleural cells in the observed fibrotic process. Further, our results suggest that caspase-1 should be considered as a therapeutic target in the management of pulmonary fibrotic disorders.

## Additional files

Additional file 1: Figure S1. Scheme of the different models used in this work. (PNG 145 kb)
Additional file 2: Figure S2. In depth subpleural collagen quantification. (PNG 1209 kb)
Additional file 3: Figure S3. Intravenous BLM injections trigger collagen accumulation mainly in the subpleural areas by D14 with overexpression of HSP47. (PNG 2604 kb)
Additional file 4: Figure S4. BLM promotes an inflammation profile of the BALF but not in the blood. (PNG 539 kb)
Additional file 5: Figure S5. BLM triggers the accumulation of NLRP3 protein in Met5A cells. (PNG 464 kb)
Additional file 6: Figure S6. Intravenous BLM induces TGF-β1 overproduction in mouse lung at D21. (PNG 425 kb)

## Abbreviations

BALF: Broncho-alveolar lavage fluid
BLM: Bleomycin
EMT: Epithelial to mesenchymal transition
FISH: Fluorescence *in situ* hybridization
HMGB1: High mobility group box 1
IL-1β: Interleukin-1β
IPF: Idiopathic pulmonary fibrosis
MMT: Mesothelio-mesenchymal transition
NLRP3: NOD-like receptor family, pyrin domain containing 3
PAI-1: Plasminogen activator inhibitor-1
PLF: Pleural lavage fluid
TGF-β: Transforming growth factor-β1
α-SMA: α-smooth muscle actin
