# Supplementary figures and images for: Pleural inhibition of the caspase-1/IL-1β pathway diminishes profibrotic lung toxicity of bleomycin
Source: Respir Res. 2016 Nov 29;17:162. doi: 10.1186/s12931-016-0475-8 (PMC5127006; doi:10.1186/s12931-016-0475-8)

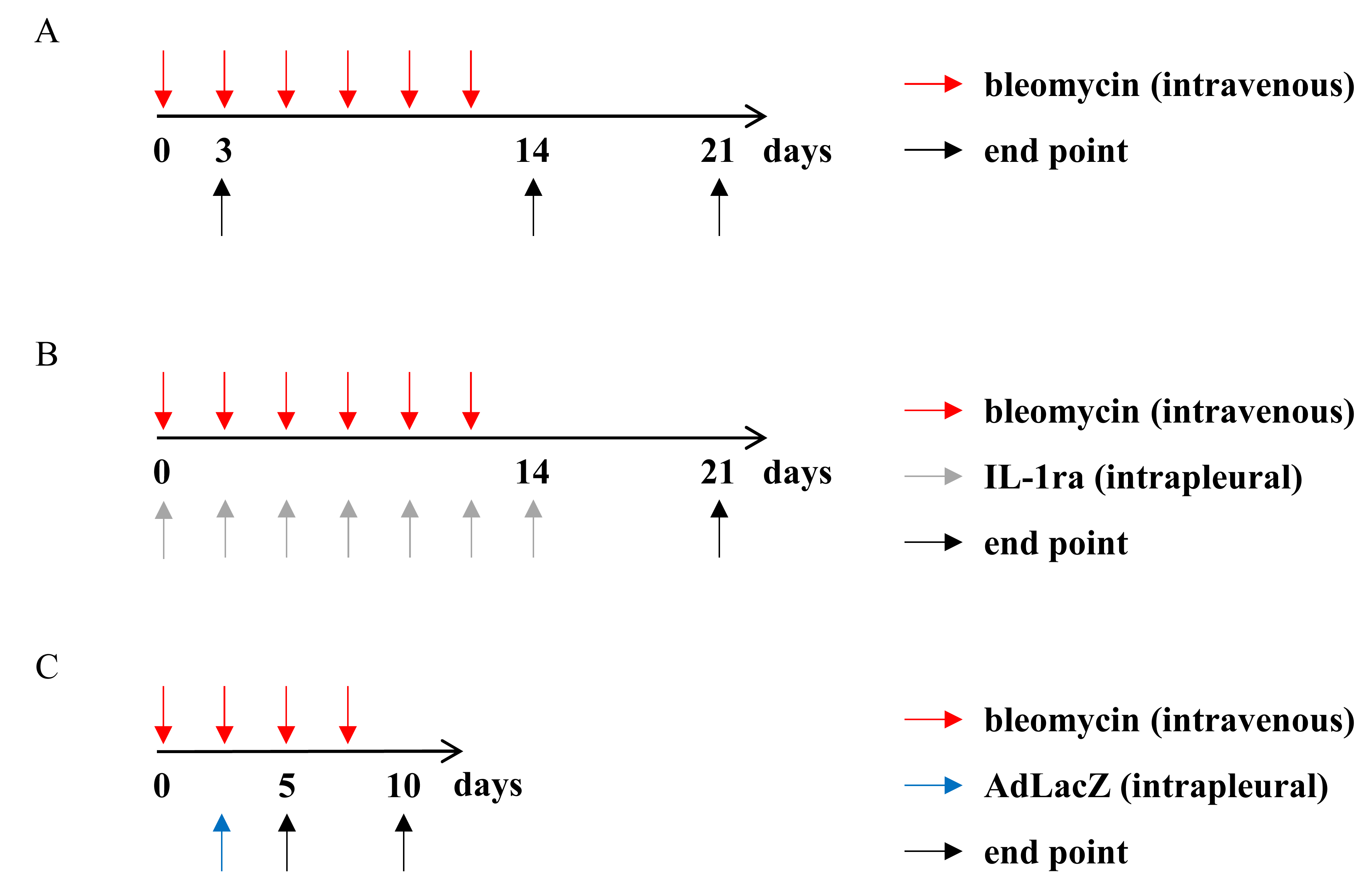

Supplement: Additional file 1: Figure S1. — Scheme of the different models used in this work. (PNG 145 kb) [file 12931_2016_475_MOESM1_ESM.png]

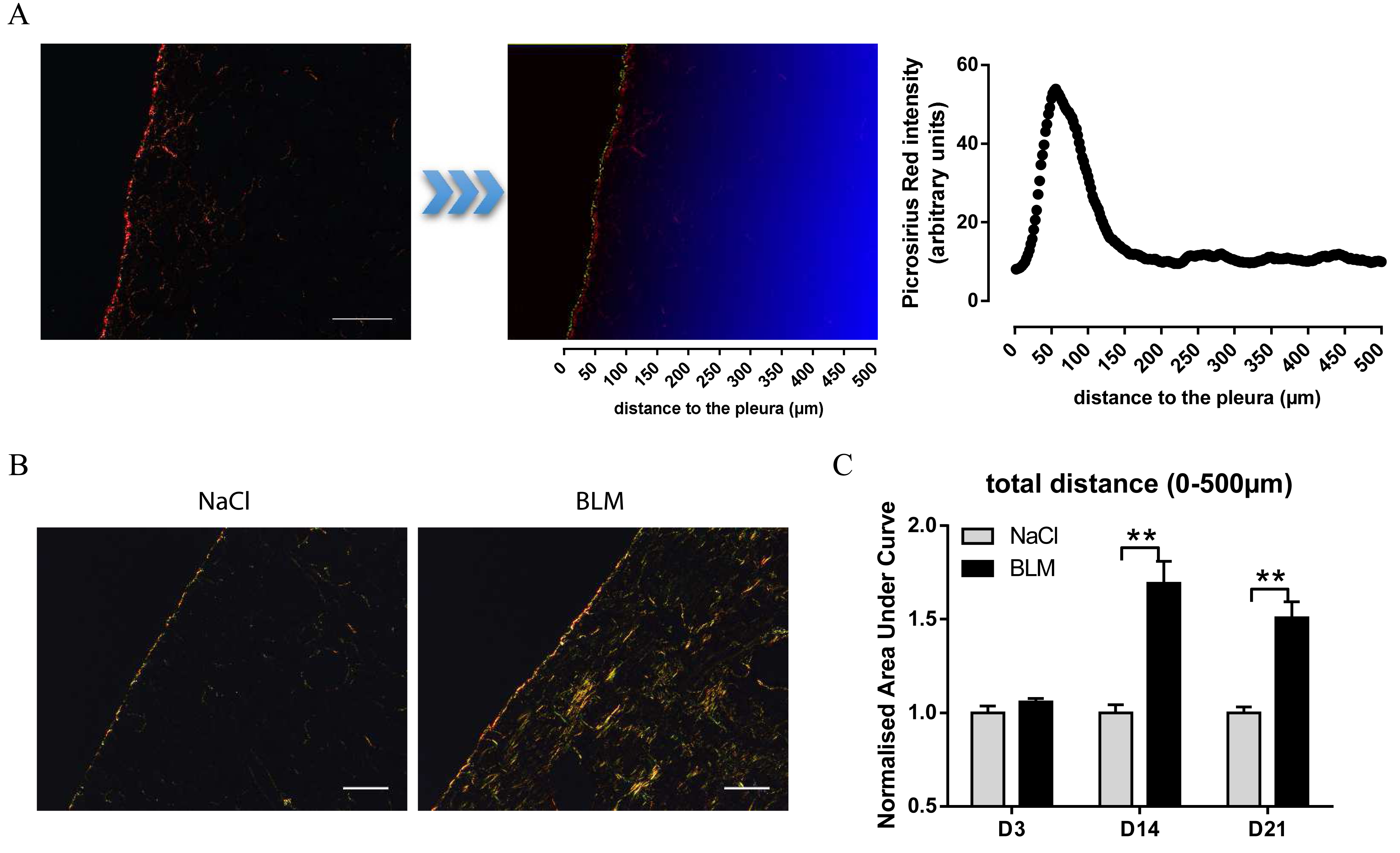

Supplement: Additional file 2: Figure S2. — In depth subpleural collagen quantification. (PNG 1209 kb) [file 12931_2016_475_MOESM2_ESM.png]

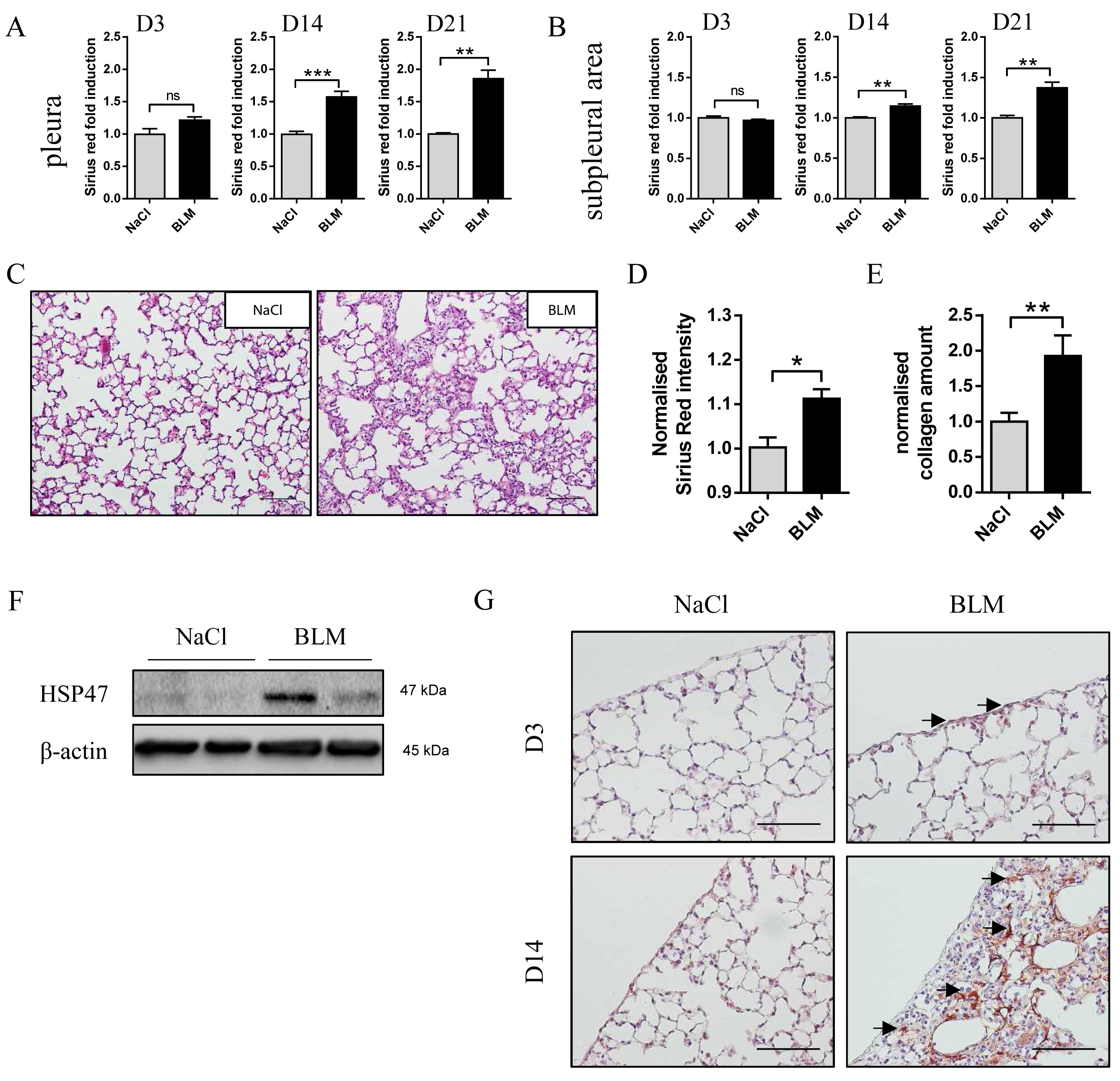

Supplement: Additional file 3: Figure S3. — Intravenous BLM injections trigger collagen accumulation mainly in the subpleural areas by D14 with overexpression of HSP47. (PNG 2604 kb) [file 12931_2016_475_MOESM3_ESM.png]

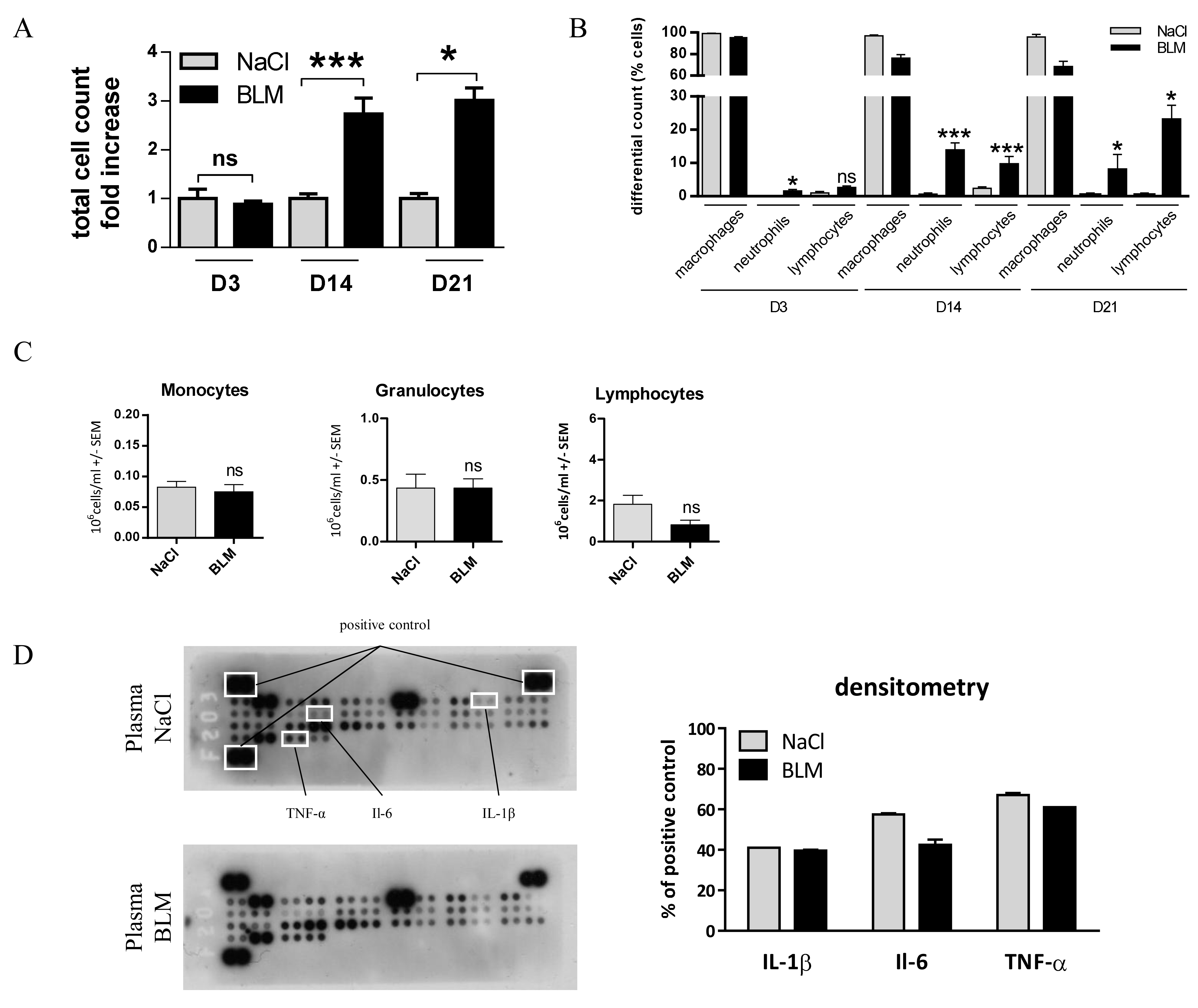

Supplement: Additional file 4: Figure S4. — BLM promotes an inflammation profile of the BALF but not in the blood. (PNG 539 kb) [file 12931_2016_475_MOESM4_ESM.png]

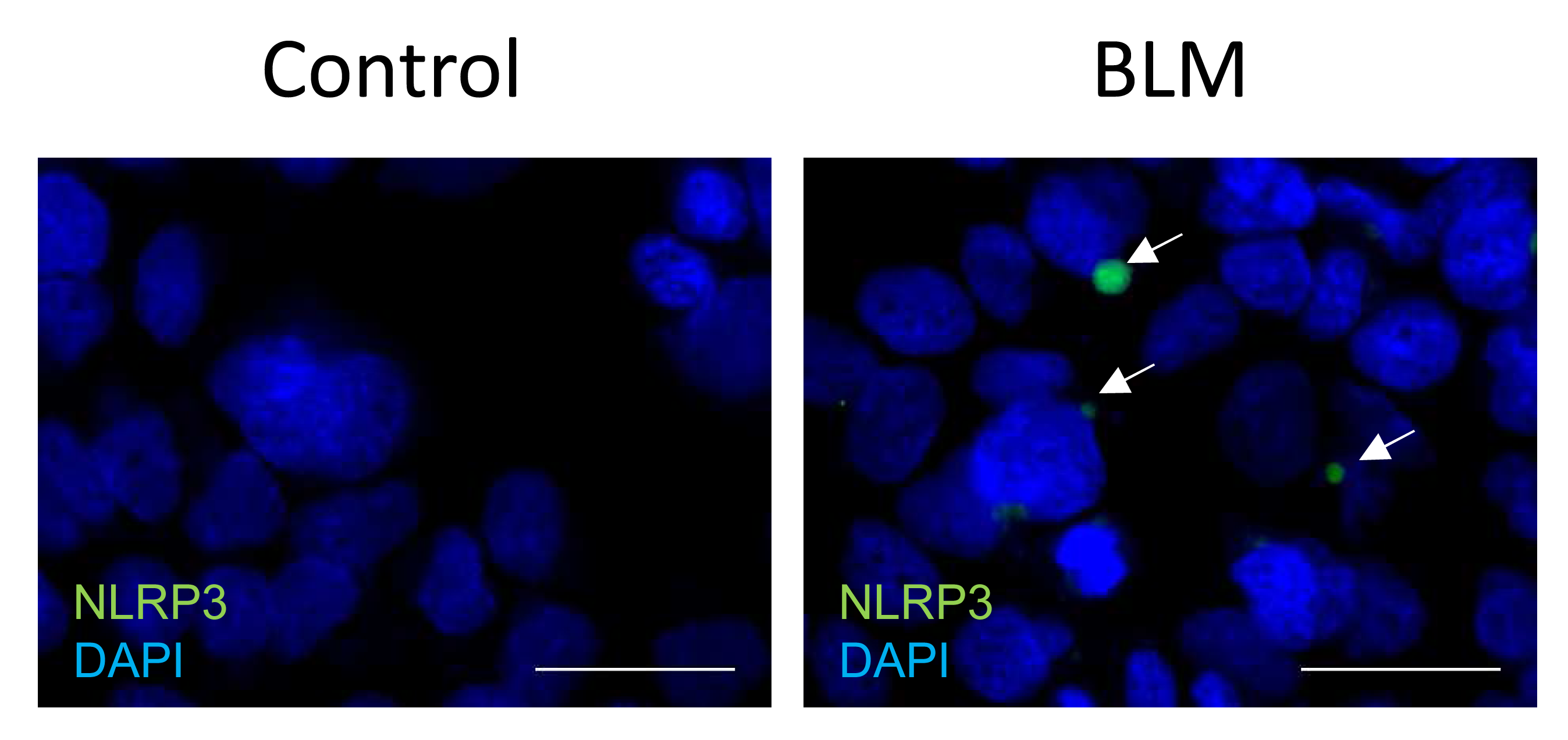

Supplement: Additional file 5: Figure S5. — BLM triggers the accumulation of NLRP3 protein in Met5A cells. (PNG 464 kb) [file 12931_2016_475_MOESM5_ESM.png]

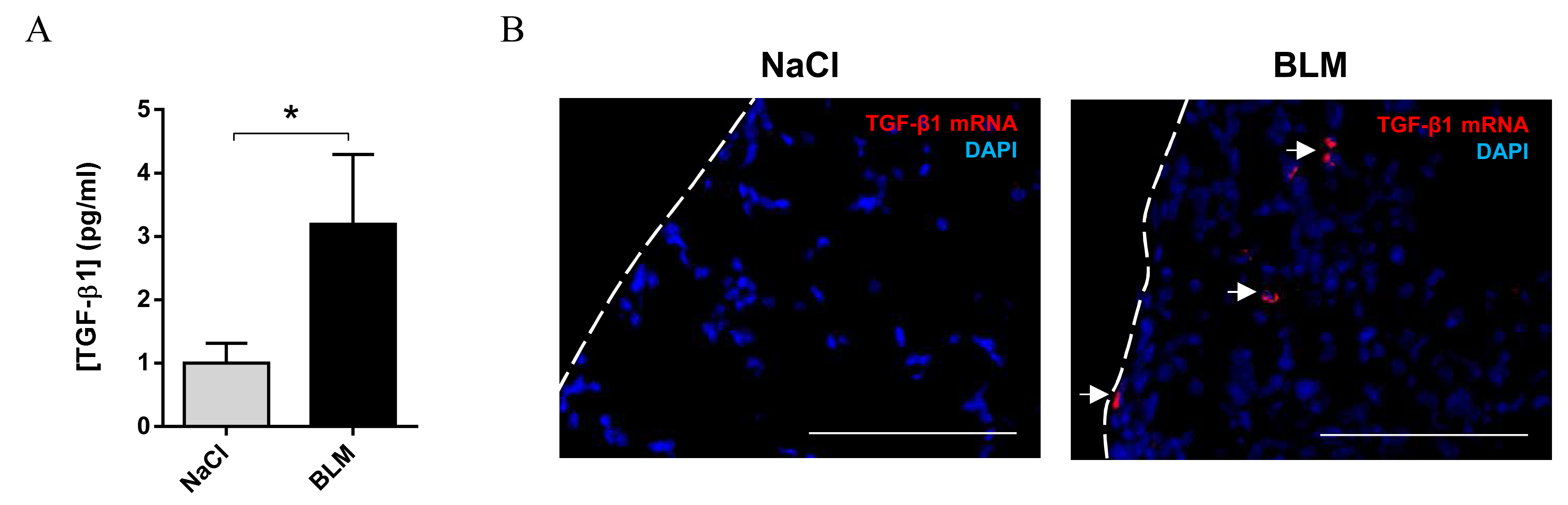

Supplement: Additional file 6: Figure S6. — Intravenous BLM induces TGF-β1 overproduction in mouse lung at D21. (PNG 425 kb) [file 12931_2016_475_MOESM6_ESM.png]
